# Patient centred care in diabetology: an Islamic perspective from South Asia

**DOI:** 10.1186/2251-6581-11-30

**Published:** 2012-12-29

**Authors:** Asfandyar K Niazi, Sanjay Kalra

**Affiliations:** 1 Shifa College of Medicine, Islamabad, Pakistan; 2 Department of Endocrinology, Bharti Hospital, Karnal, India

**Keywords:** Patient centred care, Diabetology, Islam, South Asia, Diabetes

## Abstract

Patient centred care (PCC) is a healthcare model which is sensitive towards the patients’ preferences, needs and values. Interest in the use of PCC in diabetology has heightened recently. There is a special need of the usage of PCC in Muslim communities. Six out of the ten countries with the highest prevalence of diabetes are Muslim majority countries. There are several religious and sociocultural issues specific to South Asian Muslim societies that merit the need of individualization of care for people with diabetes. Several such issues are presented in this article, along with recommendations for tackling them.

## Patient centered care (PCC)

Patient-Centered Care (PCC) has many definitions, but it is commonly defined as an approach where the health care provider is sensitive towards the patients’ preferences, needs and values [1]. It also includes the education and empowerment of patients regarding their own health and allowing them to take the decisions for their own health. Although this approach was initially developed for use in primary care and family medicine, it is now paving its way into all aspects of medicine. In this approach, the focus of the physician shifts from the disease to the patient and there is a restoration of balance in the doctor-patient relationship. Instead of a more passive role for the patient, as in the conventional healthcare models, the patient is offered a more active role in the management of his own disease and the power to take decisions is shared between the doctor and the patient.

### PCC - use in diabetology

The use of PCC has traditionally been limited to primary care. However the interest in the use of PCC in diabetology heightened after the use of this term in the 2012 guidelines of American Diabetes Association (ADA) [2]. The ADA now recommends the use of PCC in all cases of diabetes, especially type 2 diabetes. After the ADA guidelines, PCC has shifted from ‘a peripheral, psychosocial topic’ to centre stage. The ADA now recommends that the entire health care management of diabetics should revolve around PCC. They recommend giving the patients full control over their lifestyle changes, non-pharmacological management, and to a certain degree over their pharmacological management as well. The patients should not be treated out of context of their real lives and the physicians should be sensitive towards the influences of the community and the environment on the patient. In addition the patient should have full control over the level of involvement he desires to have on his own management. Some patients may prefer to take their own decisions after gaining sufficient information from their physicians while others may wish to assume a more passive role. The level of involvement desired by the patient should be evaluated on the first encounter and the physician should modify his role accordingly.

### Is this relevant to Islam?

There is a generally prevalent attitude that patients in South Asian Muslim communities are neither educated nor empowered enough to make their own decisions. They should not be given a choice regarding their own management. Instead the best choice for the patient’s health should be made by the physician on behalf of the patient. They believe that if given a choice in their own management, people with diabetes will not be able to make the best, informed choice and therefore for their own good the choices for their health should be made by the physicians themselves. They also suggest that the Muslim patients are more comfortable with assuming a more passive role in their own health care management.

### Modern Islamic socioeconomic environment

At least six of the top ten countries of the world with the highest percentage of diabetes have a majority of Muslims [3]. These Muslim majority countries are Kuwait, Lebanon, Qatar, Saudi Arabia, Bahrain, and UAE. The top ten countries accounting for the most number of diabetics worldwide include Bangladesh, Egypt and Indonesia which are Muslim majority countries and India where Islam is the second most common religion. This makes the challenges in diabetology in these countries immensely important. There is a need to focus on their specific issues if the current situation of diabetes is to be curtailed.

In the recent years, awareness and literacy has now increased in the Islamic communities. The patients feel more empowered and are keener to get involved in their own management. They are no longer satisfied with being dictated on to a management plan. The patients now demand the focus of the management to shift from the disease to their lives as a whole; with all the decisions being taken together.

These patients are fully able to make healthy and appropriate choices provided they are diabetes literate and numerate. Diabetes literacy refers to the knowledge of diabetes among the general population while diabetes numeracy refers to the ability of the patient to use numbers in daily life [4]. In the context of diabetes, these numbers are related to the blood glucose levels, time intervals and insulin doses of the patient. If a patient is diabetes literate and numerate, he should be trusted with making choices about his own health and there is no reason to consider him less capable of making his own decisions in comparison to other patients.

That being said, it is important to note that there are several religious and sociocultural issues affecting Muslim patients that are of concern to a physician practicing PCC. All of these issues need to be addressed while employing PCC for diabetics in a Muslim community. Some of these are listed below.

Common myths and challenges in the Islamic society

Gender based:

Lack of outdoor activities for women

Reluctance of female patients in visiting male doctors

Reluctance of exposure of skin by female patients to inject insulin

Lack of enthusiasm by family members in treatment of female patients

Non-acceptance of diagnosis by the female patients due to marriage issues

Diet related:

Use of oily foods and bitter vegetables to restore health

Prolonged fasting periods in Ramadan

Excessive use of honey and dates

Eating a lot of fruits in diabetes

Physical exercise related:

Lack of outdoor activities for patients, especially females

Sedentary lifestyle during Ramadan

Acceptance of diagnosis and therapy:

Use of traditional Islamic medicine

Lack of faith on modern medicine

Assuming that diabetes is a form of God’s test or punishment

Considering insulin a forbidden substance

Not using insulin injections because they may cause diseases

### Traditional Islamic medicine

Traditional Islamic medicine is not based on a biomedical model. Most of the treatments available in this form of medicine are not based on Islam, but on the sociocultural milieu of the society. Except for a handful of treatments, the roots of this form of medicine can not be traced back to Islam. It is also heavily influenced by society-, culture- and geography- specific myths and beliefs. However the current concept among the general Muslim population, especially in South Asia, is that traditional Islamic medicine is based on Islam and therefore they believe in this form strongly. In this form, the disease processes are based on myths, including supernatural entities. These traditional Muslims base their passive attitude towards health and disease on the argument that Allah has given them the disease and fighting against it would be going against His will. Contrary to this misconception, Islam preaches a proactive role towards health. Islam has always supported the use of modern medicine. It is considered a sin in Islam to harm oneself or not treat oneself if any disease has affected him.

Despite the shortcomings of the traditional model, people with diabetes may remain comfortable with this. These concepts are deep rooted in their upbringing and it is not always possible to modify them. Therefore it is wise to employ religion based analogy building in order to motivate healthy behaviour.

### Analogy building

Analogy building in health refers to the use of common examples as metaphors to promote desirable health related effects in the patients [5]. For patients with a lower educational status or rigid preformed concepts, analogy building may be especially helpful. In the case of Muslim individuals and communities, analogy building proves to be a very useful tool. It may be reasoned with them that insulin has been created by Allah and therefore it should be made use of when there is need. Not using insulin means denying the use of a gift that has been bestowed by Allah for the treatment of diabetes. It can also be said that using insulin would provide better health which would allow them to practice their religion in a more efficient manner. They may be reminded that since the Prophet (PBUH) himself used modern medicine and recommended it to the Muslims, making use of modern medicine is in essence following his footsteps.

Making use of simple and relatable examples such as the above in the counseling of the patient may overcome the challenges of low compliance based on religious or sociocultural issues.

### Traditional Islamic social structure

In traditional Islamic society, there is a strong influence of the family, community and religion on one’s lifestyle, ideas, beliefs, and practices. Many a times such influences lead to poor acceptance of the diagnosis and therapeutic measures. Treating a patient in isolation is futile because the myths and beliefs of the relatives and community would inadvertently affect the beliefs of the patient. Therefore this would lead to a suboptimal glycemic control either due to non-acceptance of diagnosis or therapy. This calls for active involvement of the family and community in diabetes care.

An example of the influence of community on individuals is the poor acceptance of polio vaccine in the Muslim communities. The opposition of polio vaccines by some Muslims has led to a rise of cases of polio in regions such as Pakistan, Nigeria and Afghanistan [6]. However the opposition has more to do with the fact that in the aforementioned areas, the community as a whole has never been educated regarding the medical importance and religious standing of the vaccine. In such cases, the best approach is to use a religious leader’s services and educate the population that such measures are supported by Islam [6].

Some other factors, especially affecting women, also need special attention. In an Islamic society, females prefer not to expose any parts of their body in public except for the head. Therefore injecting insulin in public places becomes an issue. Females with diabetes often inject insulin through the clothing, without exposing their skin. However, it should be noted that injecting insulin through the clothing may compromise the sterility of the needle and may lead to infections [7].

Female patients are also likely to be uncomfortable when being examined by a male physician. This may lead to a lack of development of an adequate doctor-patient relationship. This would affect the ability of the doctor to share information with the patient and that of the patient to share her concerns and problems with the physician. The use of a female chaperone, who may be a relative of the patient, a nurse, or a female doctor, may put the patient at ease.

Women in the Islamic societies, especially those belonging to the uneducated and the lower socioeconomic class, are more likely to spend their time indoors. This leads to reduced physical activity and sun exposure. Both of these factors may lead to an exacerbation of the metabolic syndrome. Contrary to the popular belief of Islam teaching gender discrimination, it has actually been shown that the problem is sociocultural in nature rather than religious [8]. However these sociocultural practices equally affect the care of patients with diabetes, especially in the case of female patients. Tackling this issue needs extensive counseling of the patient, her family and the community as a whole.

The traditional Islamic society, like many other conventional societies, is also affected by the local traditions and customs. These also potentially affect the acceptance and management of diabetes and therefore need to be addressed during management of the patient. Myths found in a society also adversely affect the glycemic control. Some common myths in South Asia include the use of calorie-rich foods for regaining health, avoiding the use of insulin injections to prevent diseases, and the idea that diabetes may be caused by supernatural entities.

These issues may seem overwhelming; however they can fortunately be employed in the patients’ favor. Experience has shown that involving the community in preventive and curative measures shows significantly improved patient outcomes [9-11]. Because of the closely knit traditional Islamic societies, community based programs are very efficient.

#### Involvement of the family

The family must be taken in confidence during the management of diabetes. This may include education of the spouse regarding proper cooking methods, a healthier lifestyle and dosage regimens. It may also include the education of parents in case of a minor patient or caretakers in the case of old or disabled patients. In chronic diseases, such as diabetes, the support of family and relatives is extremely important to keep the patient motivated to follow a healthier lifestyle. Making a family move as a whole on to a healthier lifestyle will not only support the patient but also lower the risk of diabetes in the family. Stress in the family or close relatives may lead to hyperglycemia. Domestic stress and marital discord are rather common precipitating factors for uncontrolled hyperglycemia. Therefore the involvement of families in the management of diabetes is warranted.

#### Involvement of the community

The general community should also be involved in spreading awareness regarding diabetes. The impact of the community leaders in a traditional Islamic society is profound. Not only can this awareness lead to prevention of risk factors and early diagnosis of diabetes but it will also help in bringing physical changes in the society. Such changes may include building and maintenance of playgrounds, jogging tracks and parks. It would also promote outdoor activities among women. The cultural conventions in the smaller cities and rural areas of India and Pakistan, especially in the Muslim community, prevent them from healthy outdoor activities. The awareness of the health benefits of exercise and outdoor activities would be especially valuable in the management of diabetes in females.

Different methods may be employed for the community education. These may include traditional street theatre, pamphlets, TV and radio. Help from the members of the community as ‘diabetes evangelists’ may be sought. The spread of awareness regarding different aspects of diabetes will also help break the myths that are extremely prevalent in the less educated societies.

#### Involvement of the religious leaders

The traditional Islamic religious leaders may not have exposure to current scientific education, and may at times be involved in the unintentional spread of harmful medical knowledge among the society, an example being the rejection of polio vaccine, but the level of influence that they have on the Muslim society is profound. Partnering with traditional Islamic scholars in spreading knowledge about diabetes may be extremely useful. This is especially true when any of the procedures or drugs involved is considered against the Islamic teachings by the general Muslim community. In these cases, the traditional Muslims are less likely to trust the information supplied to them by a physician and more likely to accept the same knowledge from a traditional scholar. Aalims usually lead the Friday congregational prayers. Before the prayer a discussion is usually kept between the Aalim and the Muslims attending the prayers. This time can be used to spread the genuine Islamic ruling on health. It can also be used to emphasize the importance of maintaining health and preventing diseases.

Many Muslims, especially those from the uneducated class, prefer traditional Islamic healers instead of doctors. The drugs prescribed by these healers have usually neither been scientifically tested nor have their safety and efficacy been demonstrated. These traditional healers may also be employed for spreading knowledge regarding diabetes. For example the traditional healers may ask the patient to continue taking the medicines prescribed by them but also get regular checkups by a physician and take the insulin prescribed by them as well.

### Ramadan fasting

Fasting is practiced by adult, healthy Muslims in the month of Ramadan. During the fast, Muslims are required to abstain from all foods and drinks, including oral and injectable drugs. Due to the nature of fasts, they may affect the glycemic control and/or compliance of the patient [12]. However there is an obvious lack of specific guidelines for managing diabetes in fasting. Asking the patients not to fast, even though may be based on scientific evidence, may not only lead to the patient fasting without telling the doctor and decreased compliance but may also offend the patient’s cultural and religious values.

The physicians should work with their patients to prepare an appropriate and individualized diet and drug plan. Some Muslim patients prefer to break their fasts with dates. In such patients, allowing them to eat a single date daily during Ramadan on the condition of avoiding other unhealthy food products, exercising and/or increasing the dose of their drugs may lead to better compliance. It may also lead to a stronger doctor-patient relationship. When the patient understands that the decision to follow a diet and drug plan has been taken after considering his concerns, he would be more eager to follow it closely. Ramadan can also be used as a time for getting the patient to adopt healthier lifestyle habits.

Recently there has developed an interest in reaching optimal blood glucose levels during Ramadan fasting. Guidelines presented for the use of oral hypoglycemic agents and insulin during Ramadan would allow the physicians to work with the patients to improve their glycemic control while still allowing the patients to fulfill their religious obligations [13,14]. This is an example of patient centered and community centered diabetology in practice.

### Financial and geographical aspects

Even though free medical treatment is available in the government run hospitals of Pakistan and India, but given the overburdened nature of these hospitals most patients prefer to visit the private sector hospitals for their health problems. Health insurance is not widely available in these countries. Therefore a big portion of the patients pay for their hospital visits and drugs out of their own pockets. The cost of care for chronic diseases like diabetes may easily fall outside the reach of the patient. In such cases, it is important to consider the patient’s social and financial issues in order to promote compliance.

Many patients may be reluctant in disclosing their financial status however the physician needs to possess this knowledge in order to prescribe the drugs that fall within the reach of the patient yet are effective. Prescribing the drugs according to each patient’s financial status may account for an enormous difference in the patient’s compliance. Similarly a doctor should attempt to minimize the number of visits a poor patient has to make to his clinic. Being unable to do so might mean losing the patient altogether.

Geographical aspects do not get the importance in the counseling and management of the patient as they deserve. Even after motivating a patient to lead a healthier lifestyle, the physician’s efforts may fail if physical resources such as gymnasiums, public parks and playgrounds are unavailable in the community. A person living in an urban area is less likely to walk to work than a person living in a rural area where private cars are less commonly used. These factors influence a patient’s lifestyle considerably. Although there is little beyond raising awareness that a physician may do to improve the geographical factors in the patient’s environment, he can suggest alternative activities which do not require these resources yet promote a healthy living.

These factors should also be kept in mind when deciding on how intensive a control on diabetes is to be used. A patient living in an area with plenty of opportunities of exercise is more likely to normalize his blood glucose levels than another patient living in area without such facilities. For such a patient, the physician can reliably assume that adequate blood glucose control can be achieved by altering the unhealthy lifestyle habits.

The geographical location of the patient also determines the food type generally consumed. It can predict whether the patient would be eating more of oily foods, meat, junk foods or vegetables. These factors ought to be considered while writing down a prescription since these determine how much control can be accomplished over the blood glucose levels through dietary and lifestyle changes.

### Islamic evidence

After discussing in detail the factors that affect the Muslim community, it should be mentioned that there are several misconceptions regarding Islam that are prevalent among Muslims. Such ideas may result from wrong interpretation of the Quranic verses or Hadiths and may adversely affect the health care seeking behavior of the Muslims. One of the most basic concepts in Islam is a person’s responsibility to his own body. According to the Islamic concepts, man does not own his body; he merely uses it by the permission of Allah. By this concept, it is required of a Muslim to protect his body from disease and harm. Not only does Islam encourages pursuing of good health, but it also makes it compulsory. Islam says that people will be held accountable for any of their actions that lead to harm or destruction of the body. In several places in the Quran and the Hadiths, this point has been highlighted. A few of the examples are cited below:

Evidence from Quran

• “Eat and drink healthy and be not prodigal” (7:31).

• Do not kill (or harm) yourselves: for verily Allah hath been to you Most Merciful! (4:29)

• And make not your own hands contribute to (your) own destruction (harm) (2:195)

Evidence from Hadiths

• “The strong believer is better and more loved by Allah than the weaker one” (Muslim)

• “The most beloved by Allah of things He is asked to grant is (Al-aafiyah) good health” (Tirmidi)

• Narrated by Usamah Bin Shareek (may Allah be pleased with him): ‘I was with the Prophet (PBUH) and some Arabs came to him asking “O Messenger of Allah, should we take medicines for any disease?” He said, “Yes, O You servants of Allah take medicine as Allah has not created a disease without creating a cure except for one”. They asked which one, he replied “old age” ’.

• The Prophet (PBUH) said: “There are two blessings which many people do not appreciate: health and leisure”.

• He (PBUH) also said: “No blessing other than faith is better than well-being”.

These references clearly show that Islam believes in a proactive role of Muslims in their health. Contrary to the concept still found in many Muslims that any disease that is inflicted upon them is a test from Allah and therefore they should suffer through it, Islam preaches its followers to utilize modern medicine for the treatment of their diseases. Islam also stresses on other healthy habits such as cleanliness, exercise, and a wholesome and healthy diet. Spreading this knowledge among Muslims may enhance their acceptance of diagnosis and treatment, and positively affect their health care seeking behaviors.

## Conclusion

Patient centered care is appropriate in all chronic diseases, especially diabetes. The Islamic patient is blessed with various resources and support systems which we have not yet tapped. These include the patient’s family, community, religion, and deeper understanding of pathogenesis of disease. At the same time, our Islamic patients may face unique challenges: finance, access to healthy food, healthy exercise facilities, access to care, and access to drugs.

Contrary to the common misconceptions, Islam supports PCC. Islam can be used a motivating factor for optimal health care. Doctors should be sensitized to this aspect of diabetology. They should develop a deeper insight regarding the sociocultural background of their patients so as to prescribe optimal PCC.

## Competing interests

The authors declare that they have no competing interests.

## Authors’ contributions

AKN wrote the first draft of the article. SK edited the article continuously. Both authors read and approved the final manuscript.
